# Supplementary figures and images for: Efficacy of a High-Iron Dietary Intervention in Women with Celiac Disease and Iron Deficiency without Anemia: A Clinical Trial
Source: Nutrients. 2020 Jul 17;12(7):2122. doi: 10.3390/nu12072122 (PMC7400798; doi:10.3390/nu12072122)

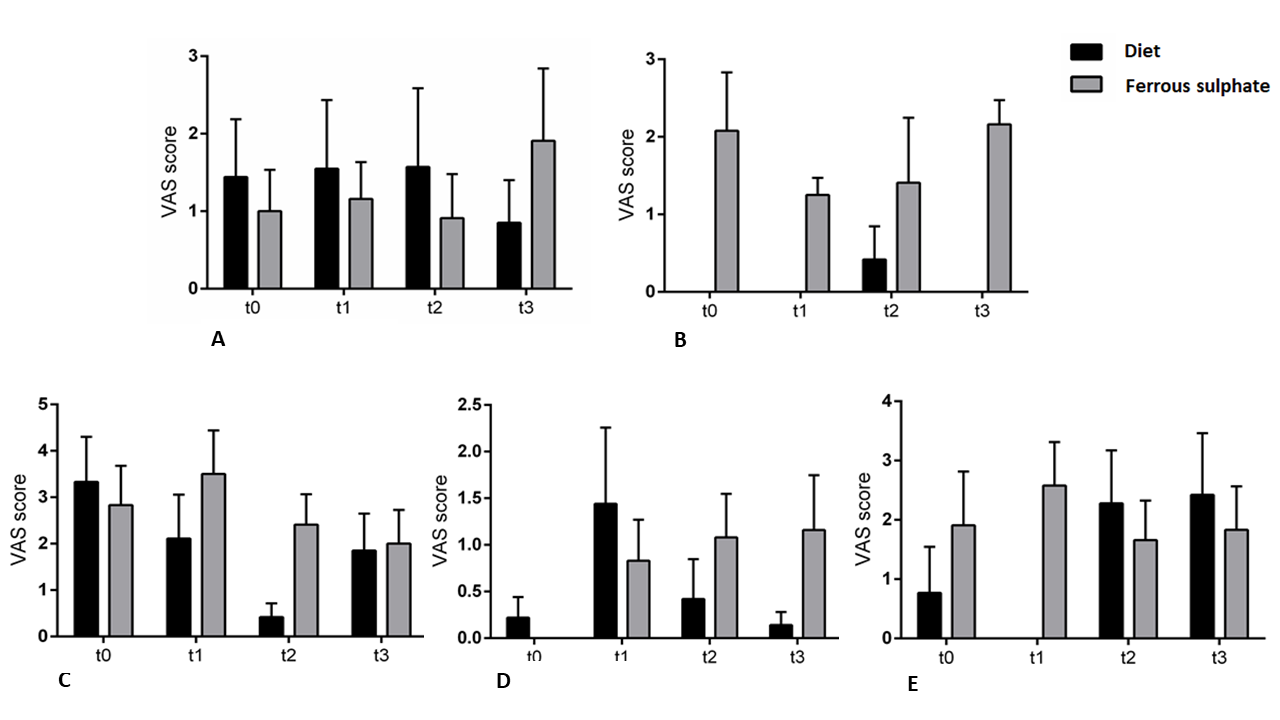

Supplement: Supplementary file 1 [file nutrients-12-02122-s001.zip › supplementary/Supp fig1.png]

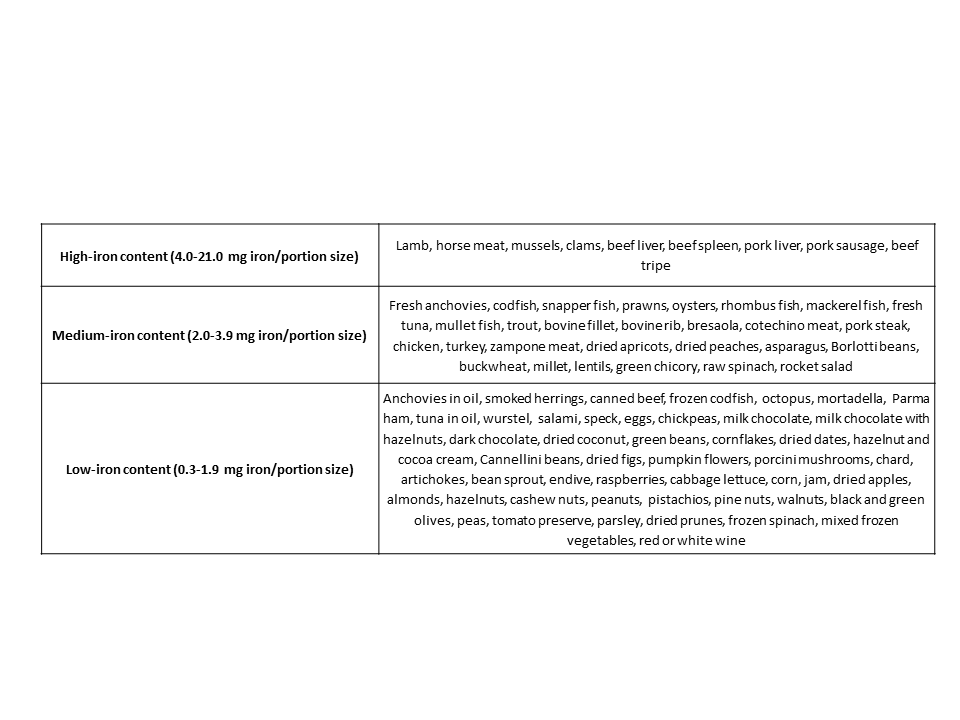

Supplement: Supplementary file 1 [file nutrients-12-02122-s001.zip › supplementary/Supp Table1.PNG]
